# Ki67, CD105, and α-SMA expression supports the transformation relevant dysplastic features in the atrophic epithelium of oral submucous fibrosis

**DOI:** 10.1371/journal.pone.0200171

**Published:** 2018-07-12

**Authors:** Amol R. Gadbail, Minal Chaudhary, Sachin C. Sarode, Shailesh Gondivkar, Satyajit A. Tekade, Prajakta Zade, Alka Hande, Gargi S. Sarode, Shankargouda Patil

**Affiliations:** 1 Department of Dentistry, Indira Gandhi Government Medical College, Nagpur, Maharashtra, India; 2 Department of Oral and Maxillofacial Pathology & Microbiology, Sharad Pawar Dental College & Hospital, Datta Meghe Institute of Medical Sciences, Sawangi (M), Wardha, Maharashtra, India; 3 Department of Oral and Maxillofacial Pathology & Microbiology, Dr. D.Y. Patil Dental College and Hospital, Dr. D.Y. Patil Vidyapeeth, Sant-Tukaram Nagar, Pimpri, Pune, Maharashtra, India; 4 Department of Oral Medicine and Radiology, Government Dental College & Hospital, Nagpur, Maharashtra, India; 5 Department of Oral and Maxillofacial Pathology & Microbiology, Modern Dental College & Research Centre, Gandhi Nagar, Indore, Madhya Pradesh, India; 6 Department of Maxillofacial Surgery and Diagnostic Sciences, Division of Oral Pathology, College of Dentistry, Jazan University, Jazan, Saudi Arabia; Navodaya Dental College and Hospital, INDIA

## Abstract

**Background:**

The grading of oral epithelial dysplasia is not possible in the atrophic epithelium of oral submucous fibrosis (OSMF). Recently, we found that features such as increased basal cell layer hyperplasia, abnormal superficial mitosis, increased nuclear-cytoplasmic ratio, increased nuclear size, and hyperchromasia represent transformation-relevant dysplastic features in the atrophic epithelium of OSMF. The presence of these features can be considered a high-risk feature for patients. However, these findings have not been tested and authenticated using markers relevant to oral carcinogenesis.

**Method:**

Paraffin-embedded tissues from 30 normal oral mucosa (NOM) and 50 OSMF were retrieved from 2008 to 2016 and subjected to immunohistochemical expression using Ki67, CD105 and α-SMA antibodies.

**Results:**

Ki67 LI showed significant increases from NOM (12.47±2.34) to LRED (23.47±3.75) to HRED (34.31±7.31) (<0.0001). Similarly, MVD was increased significantly from NOM (3.53±5.17) to LRED (27.57±12.25) to HRED (46.18±12.55) (p<0.0001). The expression of α-SMA was significantly increased from LRED (0.21±0.41) to HRED (1.13±0.56) (<0.0001). The Ki67 LI and α-SMA; MVD and α-SMA; and Ki67Ki67 LI and MVD in NOM, LRED and HRED showed a statistically significant positive correlation (P<0.0001). The increase in Ki67 LI was directly proportional to MVD and α-SMA expression from NOM to LRED to HRED (P<0.0001). The connective tissue stroma of NOM lacks α-SMA expression. Mild myofibroblast expression was noted in 4 cases of LRED (14.28%) and in 18 cases of HRED (81.81%). Moderate expression was noted only in 4 cases of HRED (22.22%).

**Conclusion:**

Ki67 LI, CD105, and α-SMA expression showed significant differences between normal, LRED and HRED. These findings further support that features such as increased basal cell layer hyperplasia, abnormal superficial mitosis, increased nuclear-cytoplasmic ratio, and hyperchromasia could be transformation-relevant dysplastic features.

## Introduction

Oral epithelial dysplasia (OED) is currently used as a tool for predicting malignant potential and determining the treatment strategy for oral potentially malignant disorders (OPMDs). However, grading OED is not always possible, especially when the epithelium is atrophic. Oral submucous fibrosis (OSMF) is considered to be a disorder with a significantly higher malignant transformation rate and morbidity, which is characterized by atrophic epithelium. The epithelial thickness in OSMF is significantly lower (8.6 ± 2.9) than the control group (26 ± 4.39)[1]. Therefore, the criteria for the current grading system of OED, which is based on the thickness (height) of the cellular and architectural changes, will not be applicable for OSMF. This finding raises the question about the authenticity of previous studies that have mentioned OED in OSMF. Recently, we found that features such as increased basal cell layer hyperplasia, abnormal superficial mitosis, increased nuclear-cytoplasmic ratio, increased nuclear size, and hyperchromasia represent transformation-relevant dysplastic features in the atrophic epithelium of OSMF. Their presence can be considered a high-risk feature for OSMF patients. Thus, there is dire need to investigate such features against the various carcinogenesis-relevant molecular markers.

The growth potential intrinsic to the lesion was assessed by measuring proliferative activity in the form of growth fraction. The proliferative activity is indicated by the percentage of cells committed to the next cell cycle. The Ki67 labeling index (LI) is simply the indicator of proliferative activity. Ki67 is a specific and sensitive biomarker of cell proliferation. The Ki67 protein is present in all the active parts of the cell cycle (G1, S, G2, M phase only) [2]. Myofibroblasts are the most prominent cancer-induced host cells of the tumor microenvironment of connective tissue. Immunohistochemically, the presence of alpha-smooth muscle actin (α-SMA) demonstrates the presence of myofibroblasts [3]. Endoglin (CD105) is a receptor for transforming growth factor-β signaling, which plays a significant role in neoangiogenesis. The detection of CD105 proteins is mainly limited to endothelial cells of blood vessels [4]. More importantly, CD105 expression is minimally expressed in quiescent preexisting vessels; thus, it is a prominent marker of newly formed tumor vessels, termed tumor neoangiogenesis. It has been verified that CD105 is present in new blood vessels with greater accuracy (mainly specificity) in comparison with other pan-endothelial molecules [5].

With these aspects in mind, the present study was designed to investigate the Ki67, CD105, and α-SMA expression with respect to the current malignant transformation-relevant dysplastic features in OSMF.

## Materials and methods

This study was conducted at the Department of Oral and Maxillofacial Pathology of Sharad Pawar Dental College and Hospital, Wardha, India. The institutional ethics committee of Datta Meghe Institute of Medical Sciences approved the study protocol (DMIMS(DU)/IEC/2014-15/953). Patients provided informed written consent to have their samples and data from their medical records used in the research. Paraffin-embedded tissues of 30 normal oral mucosa (NOM) and 50 OSMF were retrieved from 2008 to 2016. The included cases of OSFM were categorized and confirmed by using clinical criteria such as intolerance to hot and spicy foods, pale-looking oral mucosa, palpable fibrotic bands and chronic progressive trismus [6,7]. We used the criteria reported by Desai et al^1^ for atrophic epithelium including an epithelial thickness ≤13 cell layers and the loss of rete ridges. The cases that showed epithelial thickness of more than 13 cell layers were excluded. The sections were cut serially into 5-μm thicknesses to evaluate the expression of Ki67, CD105 and α-SMA antigen by immunohistochemistry. The degree of mouth opening (MO) was used as a criterion for the clinical grading of OSMF as follows: Grade I: MO > 35 mm; Grade II: MO between 30 and 35 mm; Grade III: MO between 20 and 30 mm and Grade IV: MO < 20 mm [8].

### Oral epithelial dysplasia grading

The atrophic epithelium of OSMF was carefully investigated for dysplastic features under high power magnification. Based on the previously published data [9], cases showing only basal cell hyperplasia were categorized as low-risk epithelial dysplasia (LRED). Basal cell hyperplasia with two or more other transformation-relevant dysplastic features such as abnormal superficial mitosis, increased nuclear-cytoplasmic ratio, and hyperchromasia were considered high-risk epithelial dysplasia (HRED).

### Study design

All the histopathology slides and IHC slides were blinded for analysis. Two independent experienced oral pathologists performed the OED grading. The degree of agreement between them was statistically significant. Similarly, two oral pathologists who were unaware of the OED grading status of the cases performed IHC scoring of Ki67, CD105, and α-SMA. The IHC values obtained were then correlated with the normal, LRED and HRED groups.

### Immunohistochemistry

The antigen retrieval for Ki67 and α-SMA was performed by subjecting tissue sections to 0.01 M sodium citrate buffer (pH 6.0). Tissue sections were treated with Proteinase K for 5 min to retrieve the CD105 antigen. The endogenous peroxidase activity of tissue was blocked by treatment with three percent hydrogen peroxide in methanol for 30 min. Nonspecific reactions were prevented by tissue sections incubated with ten percent serum for 10 min. Pre-diluted monoclonal mouse anti-human (MMAH) α-SMA antibody [Clone 1A4; Product code (PC): IR611, Dako, Denmark (DD)], pre-diluted MMAH Ki67 antibody [clone MIB-1; PC: N1633; DD], and concentrated MMAH CD105 antibody diluted at 1:30 [Clone SN6h; PC: M3527, DD) were incubated at 25 degrees Celsius for one hour. Pyogenic granuloma was used as a positive control for α-SMA and CD105. A known hyperplastic lymph node used as a positive control for Ki67. The HRP labeled polymer anti-mouse secondary antibody (Dako EnVision System, PC: K4000, DD) was incubated at 25 degrees Celsius for 30 min. The antigen-antibody reaction was visualized by the freshly prepared substrate/chromogen solution of 3, 3’-diaminobenzidine in buffer and counterstained in Mayer’s hematoxylin.

### Immunohistochemistry scoring

Ki67 LI: In the epithelial cells, brown stained nuclei were considered positive for Ki67. The positive epithelium was examined at 100X magnification for the most heavily labelled area for K67-positive cells. In three randomly selected fields, positively stained nuclei were counted at 400X magnification with a compound light microscope. Thus, Ki67 LI was equal to the number of positive cells multiplied by 100 and divided by the total number of cells examined.

Mean vascular density (MVD): Brown cytoplasmic staining in cells of connective tissue was clearly identified as CD105-positive vascular endothelial cells. Areas devoid of inflammation were considered for selection of hot spots. An endothelial cell or a group of cells discrete from adjacent microvessels and other elements of connective tissue were designated a separate countable microvessel. The microvessel count also included single cell sprouts as separate vessels [10]. Using a Leica DM LB 2 research microscope with Leica Qwin standard software, the mean vascular density (MVD) was measured. After scanning, MVD was measured in two hotspot areas at 100X by counting CD-105-positive vessels. The MVD was measured at depth of 500 μm away from the basement membrane of the epithelium in the connective tissue.

Assessment of α-SMA-positive cells: The sections stained with α-SMA were examined at 100X followed by 400X magnification. The internal controls (blood vessels) confirmed the accuracy of antigenic expression. Irrespective of the intensity, cytoplasmic brown-stained cells for α-SMA (other than non-endothelial and non-inflammatory) were considered myofibroblasts. The method first proposed by Tuxhorn et al. [11] and adapted by Etemad-Moghadam et al. [12] was used for the evaluation of α-SMA expression. According to these authors, the immunopositive cells immediately adjacent to the epithelium were considered to be myofibroblasts. The myofibroblast expression was determined by three observers and recorded as follows: 0 = no positive cells (PC); 1 (mild expression) = 1–33% PC; 2 (moderate expression) = 34–66% PC; and 3 (intense expression) = 67–100% PC. The final score was the score agreed on by two or more than two observers.

### Statistical analysis

The obtained data were subjected to statistical analysis using SPSS 17.0 software for Windows. The Kruskal-Wallis test and Mann-Whitney U test were used for the comparison of Ki67 LI, MVD and α-SMA expression between the NOM, LRED and HRED groups and among the different grades of OSMF. The statistical significance level was p<0.05. The correlation was carried out among Ki67 LI, MVD and α-SMA expression using Pearson’s rank correlation. The statistical significance level for both Spearman’s and Pearson’s rank correlation was p < 0.01.

## Results

The ages of the patients ranged from 18 years to 65 years with a mean of 37.66 ±11.55. A male predilection was observed with a male:female ratio of 2.84:1. All OSMF patients were habituated to areca nut quid in the form of kharra (with and without tobacco and lime), with a frequency of 5 to 20 times a day. The kharra was kept for 05–20 min in the oral cavity mainly in the lower vestibule. A maximum number of patients were reported in Grade III (21; 42.00%) followed by Grade II (16; 32.00%), Grade I (07; 14.00%) and Grade VI (06; 12.00%). Out of 50 cases of OSMF, 22 (44.00%) cases were graded as HRED and 28 (56.00%) as LRED (Table 1). All the normal cases did not show any evidence of OED. Case-wise details of molecular expressions of Ki67, CD105, and α-SMA in LRED (S1 File), HRED (S2 File) and control group (S3 File) can be found at supplementary materials.

**Table 1:** Clinico-pathological details of oral submucous fibrosis patients.

Characteristics		OSMF(n = 50)
Age	Mean ± SD	37.66 ±11.55
	Range	18–65
Gender	Male	37 (74.00%)
	Female	13 (26.00%)
Duration of Habit	Mean ± SD	15.14 ± 08.32
	Range	03–35
Risk Factors	Tobacco + lime + areca nut	40 (80.00%)
	Areca Nut	10 (20.00%)
	Smoking	27 (54.00%)
	Alcohol	22 (44.00%)
	Smoking and alcohol	20 (40.00%)
Clinical Stages	Grade I	07 (14.00%)
	Grade II	16 (32.00%)
	Grade III	21 (42.00%)
	Grade IV	06 (12.00%)
Based on transformation relevant dysplastic features [9]	Low-risk	28 (56. 00%)
	High-risk	22 (44. 00%)

**Fig 1:**
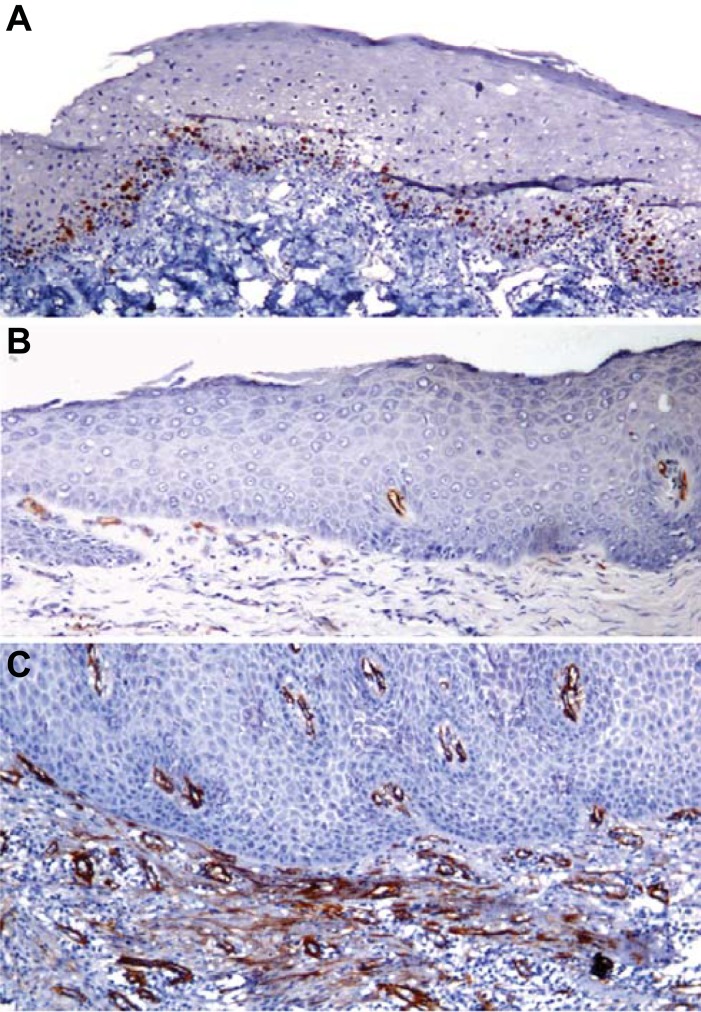
Photomicrograph of LRED Showing a) Ki67 labeling index, b) CD105 (Neoangiogenesis) and c) α-SMA Expression (100X).

**Fig 2:**
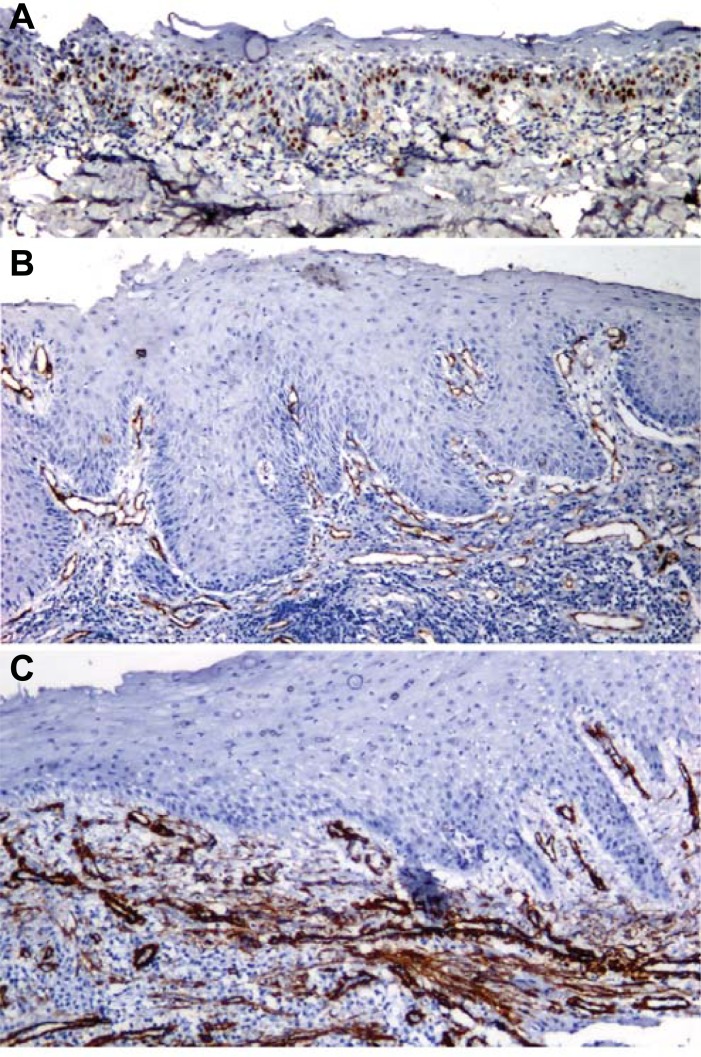
Photomicrograph of HRED showing a) Ki67 labeling index, b) CD105 (Neoangiogenesis) and c) α-SMA Expression (100X).

Ki67 LI showed a significant increase from NOM (12.47±2.34) to LRED (23.47±3.75) to HRED (34.31±7.31) (<0.0001). Similarly, MVD was increased significantly from NOM (3.53±5.17) to LRED (27.57±12.25) to HRED (46.18±12.55) (p<0.0001). The expression of α-SMA was statistically significantly increased from LRED (0.21±0.41) to HRED (1.13±0.56) (<0.0001) (Table 2) (Figs 1 and 2). The Ki67 LI and α-SMA; MVD and α-SMA; and Ki67 LI and MVD in NOM, LRED and HRED showed a statistically significant positive correlation (P<0.0001) (Table 3). The increase in Ki67 LI was directly proportional to MVD and α-SMA expression from NOM to LRED to HRED (P<0.0001). No significant differences were noted among different clinical grades of OSMF for Ki67 LI, MVD, and α-SMA expression. The connective tissue stroma of NOM lacks α-SMA expression. Mild myofibroblast expression was noted in 4 cases of LRED (14.28%) and in 18 cases of HRED (81.81%). Moderate expression was noted only in 4 cases of HRED (22.22%) (Table 4).

**Table 2:** Comparison of Ki67, MVD and Alpha-SMA expression with normal, low-risk epithelial dysplasia and high-risk epithelial dysplasia.

Biomarkers	Groups	n	Mean ± SD	Mann-Whitney U test P value
Ki67 LI	NOM (A)	30	12.47 (±2.34)	B>A, C>B P<0.001
	LERD (B)	28	23.47 (±3.75)	
	HERD (C)	22	34.31 (±7.31)	
MVD (CD105)	NOM (A)	30	3.53 (±5.17)	B>A, C>B P<0.001
	LERD (B)	28	27.57 (±12.25)	
	HERD (C)	22	46.18 (±12.55)	
α-SMA expression	NOM (A)	30	0.00 (±0.00)	B>A, (P = 0.008) C>B (P<0.001)
	LERD (B)	28	0.21 (±0.41)	
	HERD (C)	22	1.03 (±0.46)	

**Table 3:** Correlation between Ki67, MVD and alpha SMA by Pearson’s rank correlation analysis.

Markers	Correlation Coefficient	P value
Ki67 & MVD	0.945	P<0.0001
Ki67 & α-SMA	0.776	P<0.0001
MVD & α-SMA	0.788	P<0.0001

**Table 4:** Cases positive for a-SMA expression with the intensity of expression in OSMF-LRED, OSMF-HRED and NOM.

Groups	N	Positive cases	Mild expression	Moderate expression	Intense expression
NOM	30	00 (00.00%)	00 (00.00%)	00 (00.00%)	00 (00.00%)
LERD	28	04 (14.28%)	04 (100.00%)	00 (00.00%)	00 (00.00%)
HERD	22	18 (81.81%)	14 (77.77%)	04 (22.22%)	00 (00.00%)

## Discussion

Extensive research has been done on OSMF using various immunohistochemical markers to study pathogenesis, disease progression and malignant transformation. It is evident that a comparison of molecular markers has been performed with the clinical grades/stages of OMSF.

However, the comparison of molecular expression with OED grades has always been a neglected area in OSMF. Although OED plays an important role in predicting malignant transformation and developing treatment strategies, its evaluation is challenging because of the atrophic epithelium. The criteria used for the grading of OED in leukoplakia with hyperplastic epithelium will not be applicable to evaluate OED in OSMF with atrophic epithelium. Recently, our group observed that features such as increased basal cell layer hyperplasia, abnormal superficial mitosis, increased nuclear-cytoplasmic ratio, and hyperchromasia are malignant transformation-relevant dysplastic features seen in the atrophic epithelium of OSMF [9]. In the present paper, an attempt was made to compare the transformation-relevant dysplastic features with carcinogenesis-relevant biological markers.

The proliferative activity of tumor cells is an indicator of biological aggressiveness and has been reported as an important prognostic marker. Wang et al. [13] noted that Ki67 expression can play an important role in differentiating high-grade and low-grade esophageal squamous intraepithelial neoplasia. Ki67 LI was statistically significantly increased from NOM to LRED to HRED in this study. This result also supports the proposed OED grading in OSMF based on the transformation-relevant dysplastic features. Studies published to date have shown high Ki67 LI in OSMF compared to normal mucosa [14]; however, no study has performed a comparative analysis with LRED and HRED in OSMF. Therefore, a comparison to the previous literature is not possible. However, recently, it has been observed that the combination of Ki67 and p16 can differentiate between the transformation and non-transformation cases of OSMF [15]. Our study results also show that the epithelial proliferation rate is very high in OSMF patients as reported in previous studies [14,16,17]. It is quite intriguing that even after so much proliferative activity, there is atrophy of the surface epithelium instead of hyperplasia. In this regard, Sarode et al. [18] proposed that there is increased exfoliation of surface cells because of less protection from a salivary mucous gel. Therefore, even though there is increased proliferative activity, rapid exfoliation makes the epithelium atrophic.

The mucosal vasculature status in OSMF has always been studied in the context of the pathogenesis of atrophic epithelium and as a marker of malignant transformation. However, the present study mainly focuses on the comparison between angiogenesis and LRED and HRED in OSMF. We reported a significant difference in MVD and increases from NOM to LRED to HRED. These findings support OED grading based on the transformation-relevant dysplastic features. Desai et al. [1] and Sabarinath et al. [19] noted higher MVD in OSMF compared to controls using a CD34 antibody. However, in the present study, MVD was calculated using CD105 expression, which is a hypoxia-induced protein and a potential marker for neo-angiogenesis [20]. Siar et al. [21] hypothesized that the neoangiogenesis may represent an important intermediate pathological biomarker in oral precursor lesions preceding oral cancer transformation. Therefore, the assessment of MVD using CD105 expression may be useful as a predictive marker of malignant transformation in OSMF. Recently, Anura et al. [22] studied the immunohistochemical expression of CD105 in 58 biopsies of OSMF using computer-aided quantification. It was concluded that CD105 expression can be used to determine the malignant potential of OSMF [22]. Similarly, Desai et al.[1] reported that assessment of CD-34 positive blood vessels may play an important role in the malignant transformation of OSMF. Based on the correlation of angiogenesis with OED grades and the role of angiogenesis in the malignant transformation of OSMF, it can be concluded that increased neoangiogenesis using CD105 can validate the prediction the HRED in OSMF based on the transformation-relevant dysplastic features.

Tumor progression may be stimulated by myofibroblasts by stimulating the proliferation of cancer cells, attenuating the death of dysplastic cancer cells, sustaining neoangiogenesis and lymphangiogenesis, and activating the proteolysis of tumor stroma, in which the loosening of the barrier to cancer cells stimulates invasion and metastasis [23]. Myofibroblasts derived from tumor connective tissue are better promoters of tumorigenesis compared to the normal fibroblasts derived from normal tissues due to their interaction with carcinoma cells [24]. In OSMF, myofibroblast have always been linked with fibrosis and malignant transformation. Accordingly, the myofibroblast count was compared with the clinical stages (mouth opening) of OSMF. HRED showed a significantly higher expression of α-SMA compared to LRED. Thus, it can be concluded that α-SMA expression in OSMF compliments the proposed OED grading system, which is based on the transformation-relevant dysplastic features. Most studies [25–29] concluded that the myofibroblast expression can indicate an early event of the epithelial-mesenchymal interaction in disease progression and increase the risk of malignant transformation in OSMF. In this study, mild myofibroblast expression was noted in only 4 cases of LRED (14.28%) and in 18 cases of HRED (81.81%). However, moderate expression was noted only in 4 cases of HRED (22.22%). Transformed myofibroblasts in HRED in OSMF may play an active role in disease progression and may indicate the risk of malignant transformation. This finding could be due to both autocrine effects on the connective tumor stroma by triggering neoangiogenesis and proteolysis and by the paracrine effect on dysplastic epithelial cells [24]. Thus, α-SMA expression in HRED suggests the possibility that an altered keratinocyte phenotype may lead to the predisposition of malignant transformation in OSMF through epithelial-mesenchymal interactions [30].

In this study, Ki67 LI and MVD; Ki67 LI and α-SMA expression; and MVD and α-SMA expression showed statistically significant positive correlations. Increased Ki67 LI along with MVD and α-SMA expression were higher in HERD than LERD in OSMF. The increase in the proliferative activity of the epithelium supported by increased neoangiogenesis and myofibroblast expression was significantly higher in HRED than LERD in OSMF. These results suggest that all the markers together can complement the current OED grading system in OSMF. Moreover, it was suggested that the altered expression of Ki67, CD-105 and α-SMA biomarkers can indicate an event in the disturbed epithelial-mesenchymal interaction, resulting in progression towards the malignant transformation of OSMF.

Many clinicians usually don't recommend incisional biopsy for OSMF patients; because of the fibrotic nature of OSMF and post biopsy fibrosis. Results of the present study support that the recently used transformation-relevant dysplastic features [9] is useful in grading of epithelial dysplasia (low risk and high risk) for the atrophic epithelium of OSMF. The risk of malignant transformation of OSMF is 7–13% [9]. Keeping this in mind, we recommend that incisional biopsy should be mandatory for the evaluation of epithelial dysplasia to formulate the treatment plan and follow up. The results of the present study cannot be generalized to the other pathologies showing epithelial atrophy due to variations in pathogenesis process. However, a customized epithelial grading can be developed for other pathologies using our previously used study designed. [9]

## Conclusions

In conclusion, Ki67 LI, CD105, and α-SMA expression showed significant differences between normal, LRED and HRED. These findings further support that features such as increased basal cell layer hyperplasia, abnormal superficial mitosis, increased nuclear-cytoplasmic ratio, and hyperchromasia could be transformation-relevant dysplastic features. However, we recommend future studies on other carcinogenesis-relevant markers of proliferation, survival, invasion, migration and angiogenesis to investigate the legitimacy of the currently used grading system for the atrophic epithelium of OSMF. This research could further help clinicians determine the treatment and follow-up protocol for OSMF patients. The non-availability of follow-up data in the present study was the major limitation. We recommend future prospective follow-up studies on IHC markers and the currently used grading system to further strengthen our hypothesis.

## Supporting information

S1 File Molecular expression of Ki67, CD105, and α-SMA in the low risk epithelial dysplasia cases of oral submucous fibrosis. (XLSX)

S2 File Molecular expression of Ki67, CD105, and α-SMA in the high risk epithelial dysplasia cases of oral submucous fibrosis. (XLSX)

S3 File Molecular expression of Ki67, CD105, and α-SMA in the control group. (XLSX)
